# Investigating the Potential of Extracellular Vesicles as Delivery Systems for Chemotherapeutics

**DOI:** 10.3390/biomedicines12122863

**Published:** 2024-12-17

**Authors:** Alessia Brancolini, Riccardo Vago

**Affiliations:** 1 Faculty of Medicine and Surgery, Università Vita-Salute San Raffaele, 20132 Milano, Italy; 2 Urological Research Institute, Division of Experimental Oncology, IRCCS San Raffaele Scientific Institute, 20132 Milano, Italy

**Keywords:** extracellular vesicle, drug delivery, epirubicin, bladder cancer, chemotherapeutic agents

## Abstract

Background/Objectives: Standard chemotherapy is generally considered the best approach to treat many solid cancers, even accounting for severe side effects. Therefore, the development of a drug delivery system for chemotherapeutic administration could significantly improve standard chemotherapy by maintaining the cytotoxic effects of the drugs while decreasing the inherent side effects of the treatment. The aim of our study is the optimization of a loading strategy that conjugates the use of extracellular vesicles (EVs) as drug delivery carriers, by preserving their integrity, with the loading efficiency and activity maintenance of chemotherapeutics. Methods: We compared the EV loading of the chemotherapeutics epirubicin, mitomycin, methotrexate and mitoxantrone by co-incubation. Once loaded, the activity of drug-carrying EVs was tested on cancer cells and compared to that of free chemotherapeutics. Results: We defined a linear correlation between chemotherapeutics’ concentration and their absorbance at the drug-specific wavelength, which allowed the definition of a highly sensitive absorbance-based spectrophotometric quantification system, enabling the assessment of drug loading efficiency. Co-incubation of EVs and chemotherapeutics was sufficient to obtain quantifiable drug loading, and the efficacy of EV loading was drug-dependent. Epirubicin-loaded vesicles showed increased toxicity to bladder cancer cells with respect to the free chemotherapeutic. The cytotoxicity was maintained even upon 6-month storage at −80 °C of loaded EVs. Conclusion: We established an absorbance-based spectrophotometric quantification system that enables a straightforward measure of drug loading efficiency into EVs, and we demonstrated that chemotherapeutic-carrying EVs can be obtained by co-incubation, preserving and increasing drug cytotoxicity.

## 1. Introduction

The transfer of information from one cell to others plays a fundamental role in the regulation of living organisms’ functions. Extracellular vesicles (EVs) are nanosized vesicles delimited by a phospholipidic bilayer and containing soluble proteins, RNAs and DNA fragments; EVs take part in intercellular communication by connecting cells and transmitting bioactive cargo. They can be exchanged between cells in an autocrine, paracrine or endocrine manner, covering short and long distances [1]. They contribute to blood coagulation, immunosurveillance, stem cell maintenance, tissue repair and homeostasis by transferring nucleic acids, lipids and proteins to the target cells, but they are also implicated in the pathogenesis and development of multiple diseases, such as cancer, neurodegenerative diseases and cardiovascular diseases as well as the spread of viruses and pathogens [2,3,4]. EVs are produced and taken up by all types of cells and have been found in biological fluids, included blood and urine as well as seminal, uterine and cerebrospinal fluid [5,6,7,8]. EVs’ clinical application as diagnostic and prognostic biomarkers or as therapeutic cargo vehicles has raised considerable interest thanks to their intrinsic biological properties: their high biocompatibility due to being naturally present in biofluids, their cargo’s protection from degradation, and the ability to cross biological barriers granted by their nanoscale size [9]. The major challenge for the successful use of EVs in a clinical context consists of the selection or modification of EVs for precise targeting of the specific cell type/organ of interest and the presence of inherent luminal and transmembrane cargoes derived from the cell of origin, which might limit the possible exploitation of EVs as therapeutic agents [10,11]. As innate carriers of many biological elements, EVs are suitable candidates for drug delivery and have been investigated for use in therapeutic treatment upon loading with plentiful effectors [12,13,14]. Chemotherapeutics are very active molecules that kill rapidly dividing cells, but they are limited in cancer treatment due to their poor targeting ability, poor water solubility, unspecific cytotoxicity and consequent systemic side effects. EVs have been show to overcome some of these limitations, increasing drug accumulation in tumor tissues, prolonging blood half-life, reducing systemic toxicity and improving treatment efficacy [15,16,17]. EVs used for therapeutic purposes are mainly isolated from either human cell lines or primary cultures, thus limiting immunogenicity and increasing biocompatibility. Although all cell types secrete EVs for intercellular communication purposes, not all cell-derived EVs can be used as drug delivery vehicles. Indeed, strict quality standards in terms of size, production yield, cargos and surface composition are required for their use as drug delivery systems [18,19]. After selection of the EV source, isolation and characterization of the EVs and definition of the cargo, a critical issue is the delineation of a loading strategy. The mixing of EVs with the therapeutic agent, also known as passive co-incubation, is the simplest strategy for the loading of therapeutic molecules. Hydrophobic compounds are likely to easily cross the EV membrane and be loaded into EVs more efficiently than hydrophilic ones [20,21]. To facilitate cargo loading, physical and chemical methods, such as electroporation, sonication, freeze–thaw cycles, extrusion, surfactant treatment and transfection have been employed [22]. Each these methods have shown some drawback, such as damage to the vesicle structure, deformation and fusion of EV membranes or variation of surface charges and membrane protein structure [23].

In the present study, we optimized a chemotherapeutic loading strategy that combines EV structure maintenance and drug loading efficiency with a drug-specific, absorbance-based spectrophotometric quantification system. EVs loaded with epirubicin, methotrexate and mitoxantrone were tested on bladder cancer cells and their cytotoxicity compared to that of free drugs. The impact of loaded EVs’ storage conditions on chemotherapeutic activity was tested.

## 2. Materials and Methods

### 2.1. Cell Cultures

RT112 and UM-UC-3 human bladder cancer cells and human embryonic kidney (HEK) cells were maintained in RPMI-1640 (Euroclone) or DMEM (Gibco, Life Technologies Corporation, Grand Island, NY, USA), respectively, supplemented with 10% fetal bovine serum (FBS, Euroclone) and 1% penicillin/streptomycin (Gibco).

### 2.2. EV Isolation and Quantification

EVs were isolated from HEK cells as previously described [24]. Briefly, HEK cells (5 × 10^6^) were seeded in 150 mm dishes in RPMI-1640 medium supplemented with EV-depleted FBS. Cell culture media were collected 72 h after cell plating and centrifuged for 25 min at 300× *g* for cell debris removal. Supernatants were filtered through 0.22 μm filters and ultracentrifuged at 150,000× *g* for 2 h at 4 °C. Pelleted EVs were gently resuspended in PBS, and the EVs’ protein content was quantified using a BCA Protein Assay Kit (BioRad, Hercules, CA, USA). Freshly prepared samples that were not scheduled for immediate use were aliquoted and stored at −80 °C.

### 2.3. Spectrophotometric Drug Quantification

For drug quantification, the chemotherapeutics’ specific absorption–concentration linear correlations were obtained through spectrophotometric analysis using a Mithras Reader (Berthold Technologies, Bad Wildbad, Germany). Epirubicin, mitomycin, mitoxantrone and methotrexate solutions were prepared by diluting the drugs in PBS (from stock concentrations of 200 μg/mL, 100 μg/mL, 200 μg/mL and 5 mM, respectively), and serial logarithmic dilutions were plated on a 96-well plate in quadruplicate. The absorbance of each drug was then measured at 405 nm, 420 nm, 490 nm, 570 nm and 620 nm to find the drug-specific absorption wavelength; PBS was used as a blank. To exclude the possible background derived from the EVs’ light absorption at the drug-specific wavelength, 8 μg/well of HEK-derived EVs were added, and the absorbance measurement was repeated at all the wavelengths.

### 2.4. Loading of Exogenous Drugs into Isolated EVs

One hundred micrograms of HEK-derived EVs were co-incubated with chemotherapeutics for 1 h at 37 °C in PBS. Epirubicin hydrochloride (Pfizer Pharmorubicin, New York, NY, USA, 10 mg/5mL), mitomycin C (Kyowa Hakko Kirin, Tokyo, Japan, 1 mg/mL), methotrexate (Sigma-Aldrich, St. Louis, MO, USA, 100 mg powder) and mitoxantrone (Ebewe Pharma, 10 mg/5 mL) were used at final concentrations of 500 ng/μL, 250 ng/μL, 500 ng/μL and 2.5 mM. Samples were washed twice with saline solution to remove the unincorporated drug and concentrated by centrifugation through 100 KDa Amicon Ultra 2 mL Centrifugal Filters (Merck Millipore, Darmstadt, Germany). The protein content of concentrated samples was measured with a BCA Protein Assay Kit (BioRad). HEK-derived EVs resuspended in PBS in the absence of the drugs and treated following the protocol described above were used as negative controls, as were untreated HEK-derived EVs.

### 2.5. Cell Viability Assay

RT112 and UM-UC-3 bladder cancer cell lines were seeded on 96-well plates (5 × 10^3^ cells/well) and incubated with serial logarithmic concentrations of epirubicin, mitomycin C, mitoxantrone and methotrexate. For cytotoxicity evaluation of HEK-derived, drug-loaded EVs, serial logarithmic concentrations of chemotherapeutic-loaded EVs were used for cell treatment starting from 9 μg of loaded EVs for RT112 and 4.5 μg for UM-UC-3. After 72 h incubation at 37 °C, 3-(4,5-dimethylthiazol-2-yl)-2,5-diphenyltetrazolium bromide (MTT, Sigma-Aldrich, 5 mg/mL in PBS stock solution) was added to each well at a working concentration of 0.5 mg/mL and incubated 1 h at 37 °C. The supernatants were then removed, and formazan crystals were dissolved in 100 μL/well of dimethyl sulfoxide. Cell viability was assessed by measuring the absorbance at 570 nm and expressed as a percentage relative to untreated cells. Unloaded EVs and PBS were used as controls.

### 2.6. Transmission Electron Microscopy (TEM) Analysis

HEK-derived EVs were loaded with either mitoxantrone or methotrexate by simple incubation in PBS. The drug-loaded and unloaded EVs were stored for 6 months at −80 °C. Stored and freshly prepared EVs were absorbed on glow-discharged carbon-coated formvar copper grids, washed with water, contrasted with 2% uranyl acetate and air-dried. The grids were observed with a Zeiss LEO 512 transmission electron microscope. Images were acquired using a 2 K × 2 K bottom-mounted slow-scan Proscan camera controlled by EsivisionPro 3.2 software.

### 2.7. Statistical Analysis and Bioinformatic Tools

All in vitro experiments were performed at least in triplicate, and the obtained data are shown as the mean ± standard deviation (SD) or standard error (SE). Statistical significance was determined using a 2-tailed Student’s *t* test. Difference were considered significant at *: *p* < 0.05.

## 3. Results

### 3.1. Definition of Chemotherapeutics’ Concentration–Absorbance Linear Correlations

**Figure 1:**
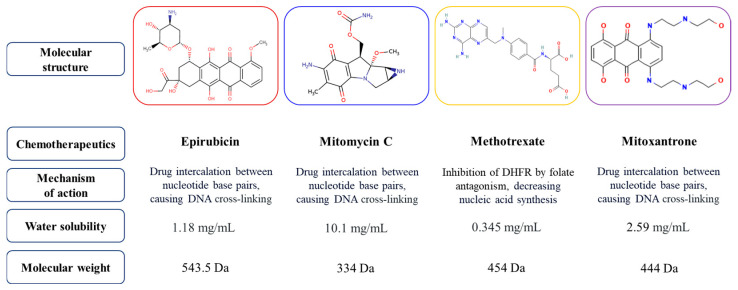
Molecular and chemical properties of chemotherapeutics used as EV payloads.

As EV payloads, four different chemotherapeutics were selected: epirubicin, mitomycin, methotrexate and mitoxantrone. All of them are currently widely used in clinics for the treatment of numerous tumors, including bladder cancer [25]. All four compounds are small molecules, characterized by different intrinsic chemical properties, such as water solubility, and mechanisms of actions (Figure 1). Importantly, all these chemotherapeutics have aromatic rings within their structures, and the presence of these chemical groups correlates with the ability of each molecule to absorb light at a specific wavelength.

**Figure 2:**
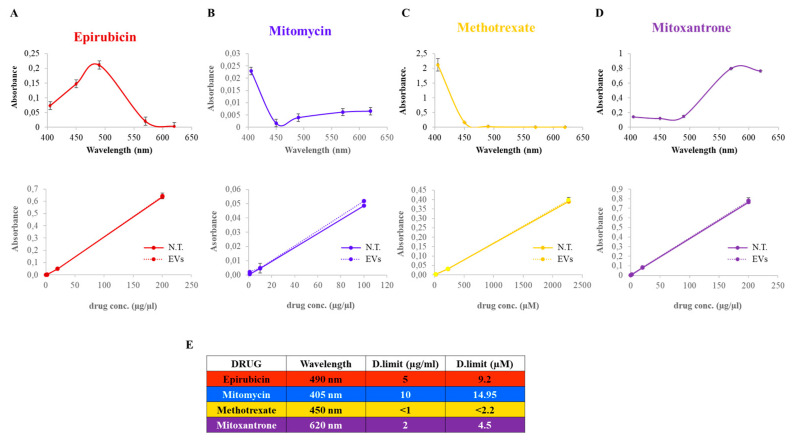
Definition of a chemotherapeutic spectrophotometric quantification system. Absorption curves of the chemotherapeutic drugs epirubicin (**A**), mitomycin (**B**), methotrexate (**C**) and mitoxantrone (**D**), measured alone (N.T.) or in the presence of EVs, expressed as absorbance versus wavelength (upper panels) or versus concentration (lower panels) at those particular chemotherapeutics’ specific absorption wavelengths, as reported in (**E**). The peak of the absorption curve is used for the definition of chemotherapeutics’ specific absorption wavelengths. Data are expressed as the mean ± SD. D.limit: detection limit.

In order to determine the drug-specific absorption wavelength of each chemotherapeutic, scalar logarithmic concentrations of each drug were measured at 405, 450, 490, 570 and 620 nm (Figure 2A–D, upper panels). Importantly, the absence of absorbance (background) at wavelengths that are not specific to the drug of interest excluded the natural color of the drug (red for epirubicin, violet for mitomycin, yellow for methotrexate and blue for mitoxantrone) as responsible of light absorbance. Then, upon identification of the drug-specific absorption wavelength of each chemotherapeutic (Figure 2E), it was possible to define the specific concentration–absorption correlation. Indeed, each compound shows a linear correlation between these two parameters (Figure 2A–D, lower panels). Furthermore, the addition of a fixed concentration of EVs to all chemotherapeutic concentrations did not alter the drugs’ specific absorbance, indicating that the EVs’ contribution was negligible and did not impede or alter the spectrophotometric drug quantification (Figure 2A–D, lower panels). This is of pivotal importance for the use of chemotherapeutics’ specific concentration–absorption correlations as a quantification system for EVs’ drug loading efficiency. The sensitivity of this quantification system is drug-specific: while epirubicin, methotrexate and mitoxantrone have a low detection limit, this is not the case for mitomycin. Indeed, only higher concentrations of the latter compound can be detected well through this quantification method (Figure 2B).

### 3.2. Cytotoxicity Evaluation of Free and EV-Loaded Chemotherapeutics in Bladder Cancer Cells

**Figure 3:**
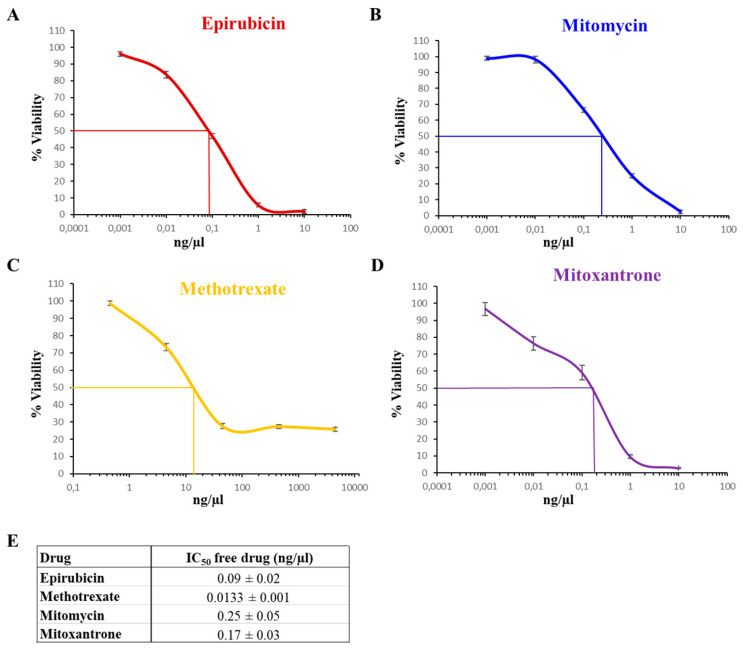
Dose–response curves of free drugs in the RT112 human bladder cancer cell line. Cell viability upon incubation with epirubicin (**A**), mitomycin (**B**), methotrexate (**C**) and mitoxantrone (**D**) was measured through an MTT assay and expressed as percentages normalized to untreated cells. Data are expressed as the mean ± SD. (**E**) Table summarizing IC_50_ values of chemotherapeutics; values are expressed as the mean ± SE.

Once we had established the drug specific spectrophotometric quantification system, the cytotoxic effect of each compound was assessed on RT112 human bladder cancer cell line. By treating cells with serial logarithmic concentrations of chemotherapeutics, it was possible to define the dose–response curve and IC_50_ value for each compound (Figure 3).

All chemotherapeutics were found to be highly cytotoxic; in particular, methotrexate was, on average, 10 times as toxic as the other compounds.

**Figure 4:**
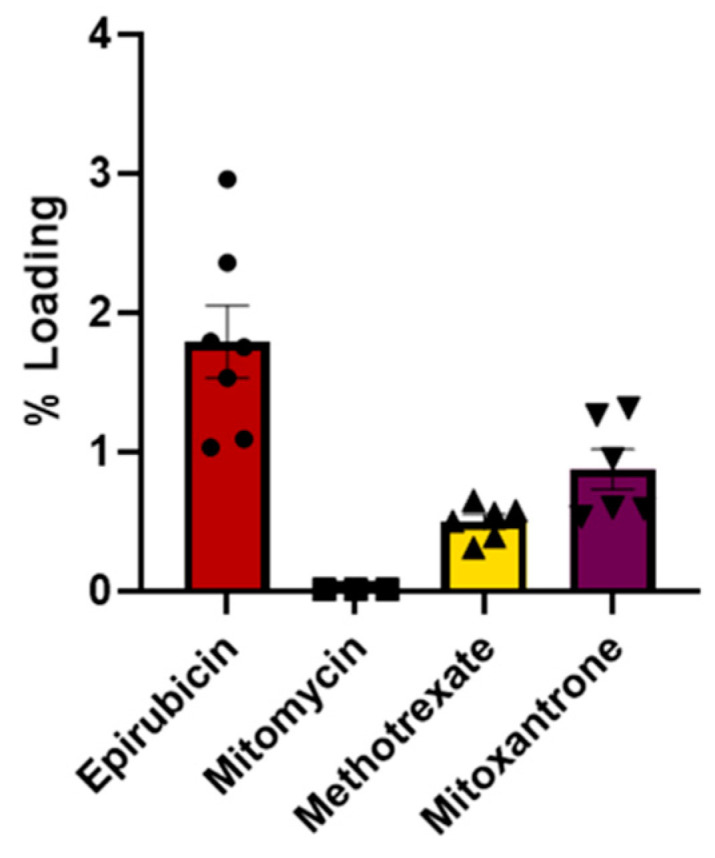
Quantification of chemotherapeutic drug loading into EVs. Chemotherapeutic loading into EVs was measured by exploiting the drug-specific spectrophotometric system described above. The percentage of loading is shown; values are expressed as the mean ± SE.

We then evaluated the efficiency of drug loading into HEK-derived EVs by passive co-incubation with chemotherapeutics. A schematic representation of the entire process, EV isolation, loading and assessment of their effect on cell viability, is shown in Supplementary Figure S1. After EV–chemotherapeutic co-incubation, non-loaded chemotherapeutic molecules were removed, and sample absorbance was assessed at the drug-specific absorption wavelength, allowing quantification of drugs loaded into EVs. Through spectrophotometric drug loading quantification, it was possible to measure epirubicin, methotrexate and mitoxantrone EV loading, while mitomycin was not detectable, its absorbance value being under the detection limit of the quantification system (Figure 4). The loading yield was estimated to be around 0.5% for methotrexate, 1% for mitoxantrone and 2% for epirubicin.

Several studies demonstrated that the EV source, isolation protocol and content, as well as the type of target cells, significantly influence vesicle toxicity and vesicle-mediated effects upon treatment [18,26]. For this reason, before assessing the cytotoxic effect of HEK-derived, chemotherapeutic-loaded EVs, we tested their intrinsic cytotoxicity in the RT112 bladder cancer cell line (Supplementary Figure S2). No toxic effects, as well as no variation of cell viability with respect to untreated cells, were detected even at the higher EV concentration in all conditions, making HEK-derived EVs suitable candidates for a drug delivery system.

**Figure 5:**
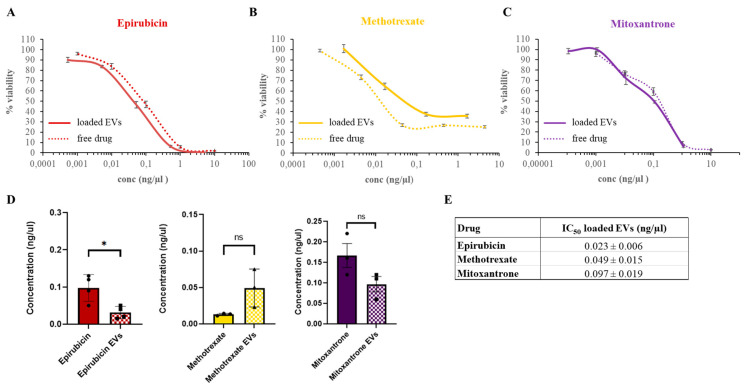
Comparison of chemotherapeutic-loaded EVs and free administered drugs toxicity on RT112 bladder cancer cells. Representative dose–response curves of epirubicin-loaded (**A**), mitoxantrone-loaded (**B**) and methotrexate-loaded (**C**) EVs (solid line) and free administered drugs (dotted line) in RT112 cells. For each drug concentration value, viability is expressed as the mean ± SD. (**D**) Comparison of IC_50_ values between chemotherapeutic-loaded EVs and free administered drugs. *: *p* < 0.05, ns: non-significant. (**E**) Table of IC_50_ values: concentrations are expressed as the mean ± SE and for each chemotherapeutic.

Then, we investigated the effect of chemotherapeutic-loaded EVs on RT112 bladder cancer cells. Serial logarithmic dilutions were used to treat cells, and cell viability was evaluated after 72 h incubation (Figure 5).

All EV-loaded chemotherapeutics show a dose-dependent increase in their cytotoxic effect on cells caused by increasing doses of EVs administered. EV-loaded mitoxantrone and methotrexate were associated with a reduction in cell viability comparable to the free drugs. EV-delivered epirubicin exerted significantly higher cytotoxicity than the drug alone. Overall, our data demonstrate that EVs can be loaded with chemotherapeutics by co-incubation and efficiently convey them to cancer cells, preserving their activity.

**Figure 6:**
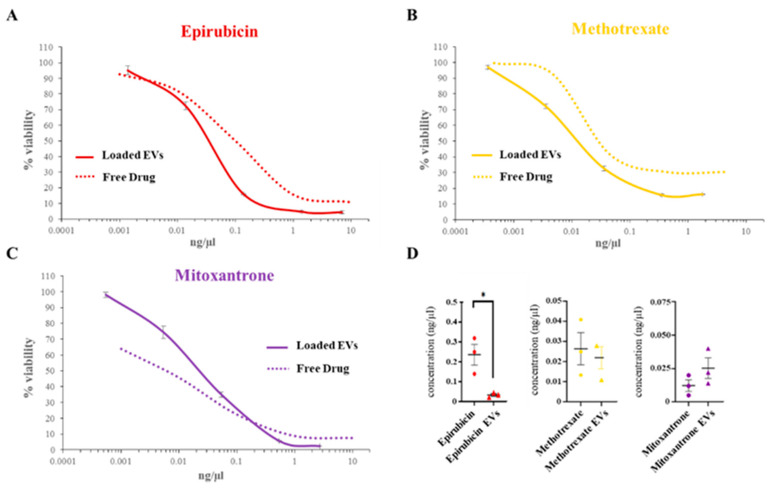
Comparison of chemotherapeutic-loaded EVs and free administered drugs toxicity on UM-UC3 bladder cancer cells. Representative dose-response curves of epirubicin (**A**), mitoxantrone (**B**) and methotrexate (**C**) loaded EVs (solid line) and free administered drugs (dotted line) on UM-UC3 cells. For each drug concentration value, viability is expressed as Mean ± SD. (**D**) Comparison of IC_50_ values between chemotherapeutic loaded EVs and free administered drugs, expressed as Mean ± SE. *: *p* < 0.05.

To confirm the results, the effect of EV-loaded chemotherapeutics was tested in another bladder cancer cell line, UM-UC-3, and compared with the effect of free drugs (Figure 6). UM-UC-3 cells showed high sensitivity to all chemotherapeutics; as for RT112 cells, methotrexate was found to be the most cytotoxic compound, being 10 times as toxic as the others. Again, the EV-delivered chemotherapeutics had comparable effects on cell viability with respect to free drugs, and EV-loaded epirubicin displayed higher cytotoxicity.

### 3.3. Effects of the Storage System on the Activity of Drug Loaded EVs

The storage of isolated EVs is a key point, especially if they are used for therapeutic purposes. Storage buffer, temperature and time are only some of the critical parameters to be set. We investigated the stability of drug-loaded vesicles under the most frequently used conditions, namely, the storage at −80 °C of PBS-resuspended EVs [27]. Methotrexate and mitoxantrone were considered as payloads and their activity upon 1- and 6-month storage at −80 °C was initially tested. The dose–response curves of the free administered drugs were performed by treating RT112 bladder cancer cells with frozen and fresh chemotherapeutics (Supplementary Figure S3). Drugs stored at −80 °C were found to be as cytotoxic as the fresh ones, indicating that the storage had not altered their activity. Accordingly, HEK-derived EVs were loaded with each of the two chemotherapeutics through simple co-incubation and frozen. As control, EVs alone were stored at −80 °C. As soon as samples were thawed, after 1 and 6 months, the EV protein content and loaded drug amount were measured (Table 1).

**Figure 7:**
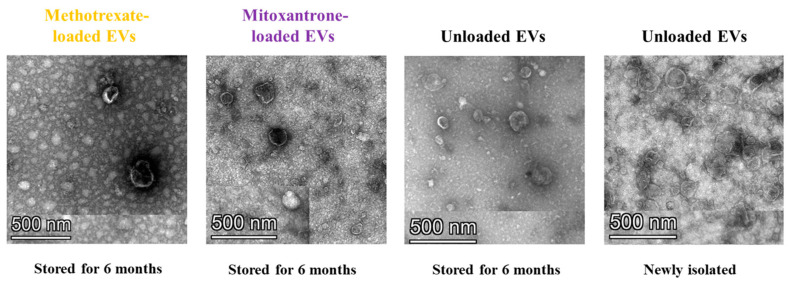
Evaluation of chemotherapeutic loaded EVs integrity upon storage. Transmission electron microscopy representative images of methotrexate and mitoxantrone-loaded EVs after 6-month storage at −80 °C. Frozen or freshly isolated, unloaded EVs are shown as controls.

After 1-month storage, the reduction of EV protein was estimated between 8 and 10%, which increased slightly after 6 months, stabilizing at 15–23%. On the other hand, the quantification of drug loading revealed a constant concentration of drugs within the vesicles over storage time. To assess the EVs’ integrity, an aliquot of each sample after 6-month storage and an aliquot of freshly isolated HEK-derived EVs were analyzed with transmission electron microscopy (Figure 7). EV structure and size remained unvaried upon storage, and no signs of aggregation or membrane damage were detectable.

**Figure 8:**
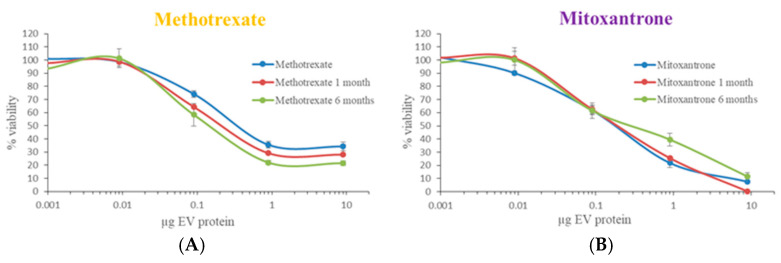
Evaluation of chemotherapeutic-loaded EVs’ cytotoxicity after storage. Cell viability curves upon administration of methotrexate-loaded (**A**) and mitoxantrone-loaded (**B**) EVs stored at −80 °C for 1 (red line) or 6 (green line) months. Freshly prepared drug-carrying EVs were used as a control (blue line). For each EV protein concentration, viability is expressed as the mean ± SD.

Finally, we tested the activity of drug-loaded EVs after 1- and 6-month storage: dose–response curves were determined in RT112 cells and compared to those of freshly loaded EVs (Figure 8 and Table 1). The cytotoxic effect of both methotrexate- and mitoxantrone-loaded EVs remained unaltered and was comparable to that freshly prepared EVs. It is noteworthy that the toxicity of frozen chemotherapeutics during storage is not associated with a protective role of the EVs in which they are loaded, since free chemotherapeutics frozen for 1 and 6 months showed the same cytotoxic effect as the fresh ones. Overall, our data indicate that these storage conditions of the loaded vesicles maintain the cytotoxic effect of chemotherapeutic-loaded EVs.

## 4. Discussion

Systemic neoadjuvant and adjuvant chemotherapy is widely used in the cancer therapy, due to its efficacy against rapidly dividing tumor cells, despite significant adverse drug reactions. For instance, epirubicin, similar to other anthracyclines, is highly cardiotoxic, and its use has been associated with cardiomyopathy and myocardial infarction even years after administration, limiting its extensive usage within therapeutic protocol [28]. However, chemotherapeutics are highly active compounds, and nanomedicine has provided a smart solution to reduce side effects: encapsulation in nanoparticles [29]. We used EVs, natural nanocarriers, to deliver epirubicin, mitomycin and methotrexate to cancer cells, preserving their activity and, in the case of epirubicin, improving its cytotoxicity. The intrinsic properties of EVs, such as cargo protection, biological barrier penetration, high biocompatibility and natural or acquired tissue/cell-targeting tropism, offer promising prospects for the improvement of therapeutic agent delivery [11]. Similarly to liposomes, which represent, thus far, the largest category of clinically approved nanoparticles, EVs are versatile drug delivery vehicles, as both the lipid membrane and internal space can be exploited for loading of hydrophobic and hydrophilic molecules, respectively. Additionally, EVs represent promising alternatives to synthetic nanoparticles, as they are naturally biocompatible due to their biological origin and can exhibit intrinsic organotropic and tumor-targeting abilities [13,30]. It has been shown that EVs have organ-specific targeting abilities that are at least partially due to integrins but most likely attributable to the interplay of several EV components [31]. Therefore, the use of EVs as drug carriers presents a promising opportunity for obtaining site-specific delivery.

Advancements in the manufacturing of classical biologics and cell therapy provide a robust framework for enhancing both upstream and downstream processes in the EV bioproduction. By optimizing these stages, it is possible to improve the efficiency, safety and therapeutic efficacy of EV-based treatments, facilitating their translation into clinical practice. Continued research in this area is crucial for overcoming existing challenges in EV production and ensuring successful therapeutic applications in medicine. Upstream processes include the selection of EV sources, expansion and harvesting; downstream processes focus on EV isolation, storage, characterization and quality control before administration [13,32].

To date, different strategies have been developed to load exogenous therapeutic cargos into purified vesicles, including passive co-incubation with or without permeabilization agents (such as saponin or detergents), sonication, electroporation and freeze–thaw cycles [12]. While passive co-incubation without permeabilization of the EV membrane is usually associated with limited cargo encapsulation, the other active loading strategies induce structural alteration and aggregation of the vesicles [33]. To define the efficiency of drug loading into EVs, we measured the absorbance of each chemotherapeutic at its specific absorption wavelength. By exploiting the natural fluorescence of the EV payloads, the spectrophotometric quantification system we developed enables an easy, low-cost and precise quantification of the loading efficiency into EVs of different chemotherapeutics commonly used in cancer therapy. During the analysis, loaded EVs do not need to be lysed, fixed or discarded, and their integrity is preserved. This approach can be extended to other therapeutic molecules characterized by intrinsic fluorescence or induced by tagging responsive domains. To be a good delivery system, the carrier should not have a cytotoxic effect on recipient cells. We showed that our EVs isolated from HEK cells do not have any toxic effect per se on recipient cells.

The nature of the therapeutic cargo used as an EV payload is a key feature for the definition of the optimal loading strategy to be employed [34]. Although hydrophilic compounds are generally considered challenging payloads to encapsulate within EVs though passive co-incubation [35], in this study, we showed that water solubility is not the only parameter to be considered to determine which vesicle loading strategy should be used. Indeed, despite the high hydrophobicity of methotrexate, it showed similar loading efficiency to mitoxantrone, while epirubicin had higher loading efficiency. Only epirubicin-carrying EVs caused a statistically significant reduction in the IC_50_ value compared to the free administered drug in both RT112 and UM-UC-3 bladder cancer cells, while methotrexate and mitoxantrone loading did not show any advantage, suggesting that it is mediated by a cell-type-independent mechanism. Even if methotrexate- and mitoxantrone-carrying EVs showed similar cytotoxic effects to the free drugs, it is important to consider that a delivery system could reduce chemotherapeutic-associated side effects and improve the specific therapeutic index in vivo. For instance, in doxorubicin (Dox) treatment regimens, one of the main side effects is cardiac injury. In an orthotopic triple negative breast cancer mouse model, inhibition of tumor growth and reduced cardiotoxicity have been achieved in Dox-loaded EVs compared to the free drug, indicating that EVs are safe and effective drug delivery carriers for targeted tumor therapy [36].

For the development of an efficient EV-based drug delivery system, the chemotherapeutic-loaded vesicles should be stable and maintain their cytotoxicity upon appropriate storage [27,37]. For this reason, we tested loaded vesicles integrity, chemotherapeutic loading and drug loaded EVs cytotoxicity upon 6-month storage at −80 °C, recommended as the optimum long-term storage with minimal damage to EVs and their molecular cargo [38]. No substantial changes were visible in vesicle morphology or size compared to fresh isolated HEK-derived vesicles. After 6 months of storage, a decrease in the vesicular protein content was measurable, in accordance with previous studies [27], suggesting that the storage conditions can slightly damage the protein component of the sample over the months. Moreover, the loading efficiency of the vesicles remained unvaried over time, as well as the cytotoxic effect of the stored vesicles on bladder cancer cells, indicating the lack of damages to the drug structure and the maintenance of loaded-vesicle stability over time.

## 5. Conclusions

We established a spectrophotometric quantification system that enables a straightforward measure of drug loading efficiency into EVs by exploiting the presence of aromatic groups within the therapeutics payloads used. Passive co-incubation of epirubicin, methotrexate and mitoxantrone with EVs was sufficient for efficient drug loading and was cytotoxic to cancer cells with comparable or higher activity than the free drugs. A subsequent in vivo evaluation will be needed to confirm the cytotoxic effects of chemotherapeutic-loaded EVs and to evaluate side effects (hepatic and cardiac damage) compared to free administered drugs.

## Figures and Tables

**Table 1:** Quantification of EV protein content expressed as a reduction percentage, drug loading yield as a percentage and cytotoxicity as the IC_50_ after 1- and 6-month storage at −80 °C.

	Protein Reduction %		Loading %			IC_50_ Drug-Loaded EVs (ng/µL)		
Treatment	1 Month	6 Months	Fresh	1 Month	6 Months	Fresh	1 Month	6 Months
Methotrexate	8.93	15.49	0.29	0.29	0.27	0.025	0.022	0.018
Mitoxantrone	10.89	23.14	0.35	0.34	0.30	0.019	0.020	0.030

## Data Availability

All data generated or analyzed during this study are included in this published article and its Supplementary Information files.

## Supplementary Materials

The following supporting information can be downloaded at: https://www.mdpi.com/article/10.3390/biomedicines12122863/s1, Figure S1: Schematic workflow of EV loading with chemotherapeutics. EV loading with drugs was performed on freshly isolated HEK293-derived EVs. EV protein content was measured and serial logarithmic concentrations were used to treat RT112 bladder cancer cells; Figure S2: Evaluation of inherent cytotoxicity of HEK239-derived EVs to the RT112 bladder cancer cell line. RT112 cell viability curves upon treatment with HEK293-derived EVs, either loaded or subjected to a purification procedure mimicking the protocol of chemotherapeutic loading (washed EVs). Cell viability is expressed as the mean ± SD; Figure S3: Evaluation of chemotherapeutics’ cytotoxicity over time upon storage at −80 °C. Viability of RT112 bladder cancer cells upon treatment with fresh, 1-month-old and 6-month-old preparations of methotrexate (left) and mitoxantrone (right) measured through an MTT assay. Viability percentages were normalized to untreated cells and are expressed as the mean ± SD.
