# Supplementary material for: Investigating the Potential of Extracellular Vesicles as Delivery Systems for Chemotherapeutics
Source: Biomedicines. 2024 Dec 17;12(12):2863. doi: 10.3390/biomedicines12122863 (PMC11673336; doi:10.3390/biomedicines12122863)
Supplement: Supplementary file 1 [file biomedicines-12-02863-s001.zip › biomedicines-3347072-supplementary.pdf]

Supplementary Material: Investigating the potential of extracellular vesicles as delivery systems of chemotherapeutics

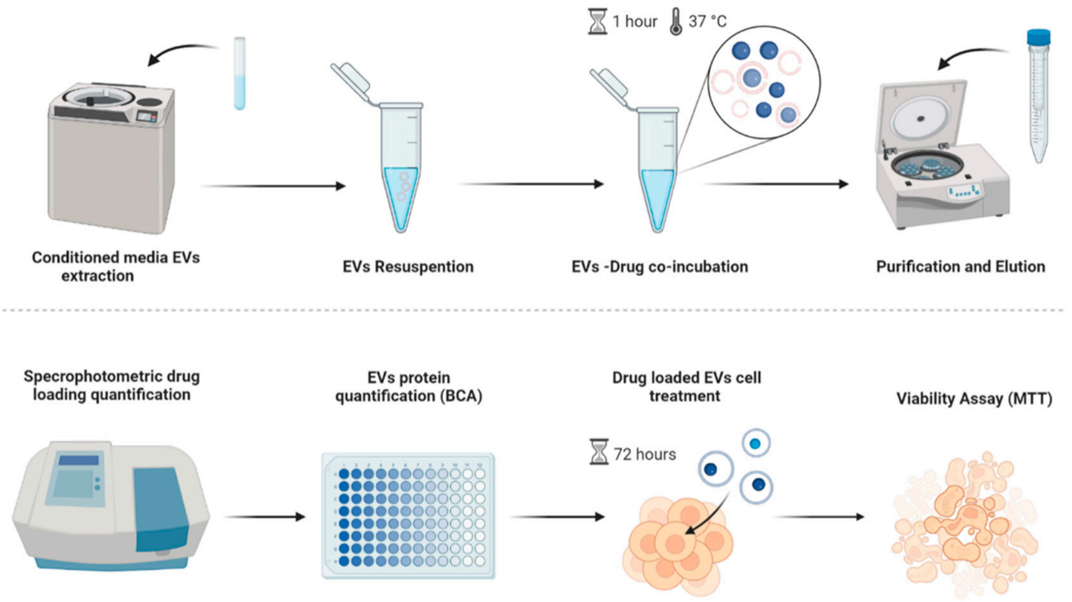

Supplementary Figure S1. Schematic workflow of EV loading with chemotherapeutics. EVs loading with drug was performed on freshly isolated HEK293 derived EVs. EV protein content was measured and serial logarithmic concentrations were used to treat RT112 bladder cancer cells.

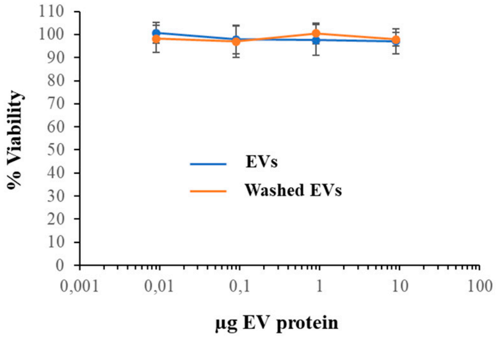

Supplementary Figure S2. Evaluation of inherent cytotoxicity of HEK239 derived EVs on RT112 bladder cancer cell line. RT112 cell viability curves upon treatment with HEK293 derived EVs, or previously subjected to purification procedure mimicking the protocol of chemotherapeutic loading (washed EVs). Cell viability is expressed as Mean ± SD.

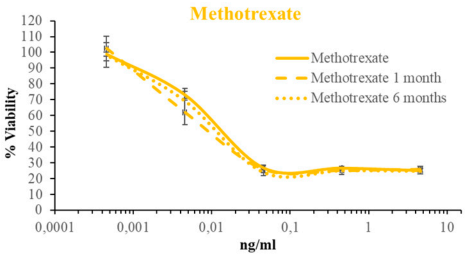

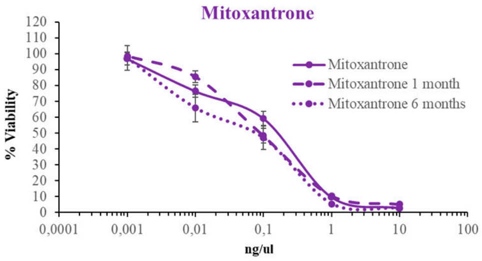

Supplementary Figure S3. Evaluation of chemotherapeutics cytotoxicity over time upon storage. Viability of RT112 bladder cancer cells upon treatment with fresh, 1- and 6-month stored methotrexate (left) and mitoxantrone (right) measured through MTT assay. Viability percentages was normalized on untreated cells and expressed as Mean ± SD.
